# NADPH Oxidase 1 and Its Derived Reactive Oxygen Species Mediated Tissue Injury and Repair

**DOI:** 10.1155/2014/282854

**Published:** 2014-01-19

**Authors:** Xiu-Jun Fu, Ying-Bo Peng, Yi-Ping Hu, You-Zhen Shi, Min Yao, Xiong Zhang

**Affiliations:** ^1^Department of Burns and Plastic Surgery, No. 3 People's Hospital, Institute of Traumatic Medicine, School of Medicine, Shanghai Jiao Tong University, Shanghai 201900, China; ^2^Department of Burns, The Fourth Hospital Affiliated to Jinan University, Guangzhou 51022, China; ^3^Department of Dermatology, Wellman Center for Photomedicine, Harvard Medical School, Massachusetts General Hospital, Boston, MA 02114, USA; ^4^Department of Burns and Plastic Surgery, Ruijin Hospital, School of Medicine, Shanghai Jiao Tong University, Shanghai 200025, China

## Abstract

Reactive oxygen species are mostly viewed to cause oxidative damage to various cells and induce organ dysfunction after ischemia-reperfusion injury. However, they are also considered as crucial molecules for cellular signal transduction in biology. NADPH oxidase, whose only function is reactive oxygen species production, has been extensively investigated in many cell types especially phagocytes. The deficiency of NADPH oxidase extends the process of inflammation and delays tissue repair, which causes chronic granulomatous disease in patients. NADPH oxidase 1, one member of the NADPH oxidase family, is not only constitutively expressed in a variety of tissues, but also induced to increase expression in both mRNA and protein levels under many circumstances. NADPH oxidase 1 and its derived reactive oxygen species are suggested to be able to regulate inflammation reaction, cell proliferation and migration, and extracellular matrix synthesis, which contribute to the processes of tissue injury and repair.

## 1. Introduction

The general view of the primary role of reactive oxygen species (ROS) in biology is to cause oxidative damage to organs and tissues suffering ischemia-reperfusion injury [1–3] and inactivate and clear microorganisms through respiratory burst of phagocytic cells [4]. High concentration of hydrogen peroxide is used clinically for wound disinfection, which might not be beneficial for overall wound healing because of the oxidative damage to host tissue in addition to bacteria [5, 6]. However, low concentration of ROS regulates intracellular signal transduction pathways by redox-dependent mechanisms, which facilitates the process of tissue repair [6]. As signal transduction molecules, ROS are controlling a large array of biological processes including the regulation of organ development and cell growth and the response to environmental stimuli [4]. In the process of tissue injury and repair, ROS has both detrimental and beneficial roles through regulating cell damages and promoting cell proliferation and migration.

One of the most important sources of intracellular ROS is the enzyme NADPH oxidase (Nox), which is the only mammalian enzyme dedicated to ROS generation. NADPH oxidase enzyme complex, formed by Nox and other cytosolic subunits, catalyzes the production of ROS from molecular oxygen. The Nox family has been extensively investigated in many cell types especially phagocytes [7]. The ROS and their oxidants are critical for bacteria and necrotic tissue purging by phagocytes. And the deficiency of Nox extends the process of inflammation and delays tissue repair, which causes chronic granulomatous disease (CGD) in patients [8]. NADPH oxidase was further found in extensive cell types such as epithelial cells, fibroblasts, and vascular endothelial cells [9]. A large number of evidence suggest that NADPH oxidase contributes to the initiation and development of many physiological and pathophysiological events, including thyroid hormone production in the thyroid gland, ischemia-reperfusion injury in multiple organs, septic shock, obesity, cancer, neuronal degeneration, and cardiovascular diseases, as well as vascular diseases [10–12]. Based on these, Nox and its derived ROS are suggested to play an essential role in wound repair and regeneration, through modulating inflammation reaction, cell proliferation and migration, and extracellular matrix synthesis and deposition [13, 14].

Nox1 as the first discovered homologue of the catalytic subunit of the superoxide-generating NADPH oxidase of phagocytes is expressed in multiple organs and various cell types, especially in colon epithelial cells and vascular smooth muscle cells [15]. In addition to its constitutive expression in a variety of tissues, Nox1 is induced to increase expression in both mRNA and protein levels under many circumstances such as proinflammatory factors and growth factors stimulation, ultraviolet (UV) radiation, hypoxia, and mechanical injury [16–19]. This review will focus on the possible roles Nox1 plays in the process of tissue injury and repair mainly through regulating the function of repair cells, namely epithelial cells, fibroblast cells, and endothelial cells and smooth muscle cells.

## 2. Nox Family and Nox1

**Figure 1:**
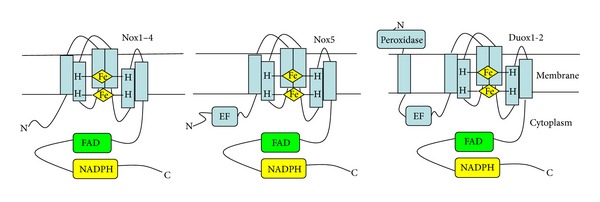
Structural differences among mammalian Nox homologues. Nox1–5 share six highly conserved transmembrane domains, while Duox1 and Duox2 have an additional N-terminal transmembrane domain. Four conserved histidines that bind two hemes between the third and fifth (fourth and sixth in Duox) of the transmembrane domains provide an oxygen binding site. The cytoplasmic C-terminus contains domans for binding of the substrate NADPH and the cofactor FAD. An additional N-terminal extension containing Ca^2+^-binding EF hands exists in both Nox5 and Duox, allowing for Ca^2+^ activation. Duox also has an extracellular peroxidase homology domain at the N-terminus.

In mammalian, the Nox enzymes can be divided into three subfamilies: one containing Nox1–Nox4 (the Nox1–Nox4 subgroup), which form a heterodimer with p22^phox^; the Nox5 subfamily; and the Duox subfamily (Figure 1). All Nox family members are transmembrane proteins that transport electrons across biological membranes to reduce oxygen to superoxide. In accordance with this preserved function, there are conserved structural properties of Nox enzymes that are common to all family members. Starting from the COOH terminus, these conserved structural features include an NADPH-binding site at the cytoplasmic COOH terminus, a FAD-binding region in proximity of the NADPH-binding site, six conserved transmembrane domains, and four highly conserved heme-binding histidines in the third and fifth transmembrane domains [7, 20]. A long intracellular NH_2_ terminus containing a Ca^2+^-binding EF hand domain is present in Nox5 and Duox proteins, distinguishing them from Nox1–4. And given the additional NH_2_-terminal transmembrane domain, the histidines are in the fourth and sixth transmembrane domains in Duox proteins [7].

Nox1 was the first homolog of Nox2 to be described. The number and the length of the exons of the Nox1 genes are virtually identical to Nox2. And at the protein level, similarly, there is a high degree of sequence identity (57%) between Nox1 and Nox2 [15]. Like Nox2, Nox1 is broadly expressed in a variety of cell types, including vascular smooth muscle cells, endothelial cells, uterus, placenta, prostate, osteoclasts, and retinal pericytes, as well as in several cell lines such as the colon tumor cell lines Caco2 and HT29 and the pulmonary epithelial cell line A549 [7, 9, 15]. However, it is most highly expressed in colon epithelium. Two cytosolic subunits are necessary for Nox1 to generate superoxide. One is Nox Organizer 1 (NoxO1) having the same role as p47^phox^, while the other is Nox Activator 1 (NoxA1), which is similar to p67^phox^. In addition to these two cytosolic subunits, Nox1 as well as Nox2 depends on the membrane subunit p22^phox^ [21, 22]. However, the dependence of Nox1 on subunit p22^phox^ is not so strict as its analogues Nox2 and Nox3. Like Nox2, the activation of Nox1 also depends on the Rac1, which provides a major trigger for Nox1-dependent ROS generation [23].

**Figure 2:**
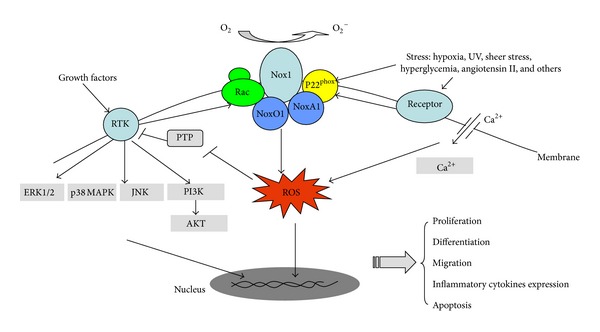
Nox1 modulates intracellular signaling. Nox1 can be activated by a diverse array of stimuli, such as the binding of growth factors to their receptor tyrosine kinases (RTK) and the stimulation by agonists such as angiotensin II. ROS produced by activated Nox1 oxidize the cysteine residue of protein tyrosine phosphatases (PTP), inactivate these enzymes, and lead to enhanced activation of MAPK system and PI3 K. ROS may also interact with intracellular Ca^2+^ by enhancing the entry of Ca^2+^ through cell membrane. The activated intracellular signals may further activate Nox1 or other Nox homologues, causing additional increasing of ROS. All these mechanisms may be involved in regulating cell proliferation, differentiation, apoptosis, and migration, and angiogenesis, which are crucial components of tissue injury and repair.

ROS from Nox1 and other Nox isoforms can achieve the regulation of cell proliferation, differentiation, survival, apoptosis, metabolism, and migration through redox-sensitive cysteine residues. A very common targeted molecule by ROS inside cell is protein tyrosine phosphorylations (PTPs), which controls the phosphorylation of tremendous proteins involving cellular signal transduction [24]. Although the precise mechanism is presently unknown, the mitogen-activated protein kinase (MAPK) system and phosphoinositide 3-kinase (PI3K) activated by the Nox family including Nox1 are shown in numerous studies (Figure 2) [24, 25]. Nox isoforms derived ROS has also been suggested to regulate some ion channels such as potassium channel and membrane and intracellular calcium channels. And these might happen through the ROS-sensitive signaling systems. Interestingly, Nox enzymes may reversibly be activated by the changing of intracellular calcium [26]. In addition, abundant evidences indicate that Nox-dependent ROS influence the expression of multiple genes, including chemotactic factors, inflammatory factors, and growth factors [27, 28].

## 3. The Activation of Nox1 in Epithelial Cells and Wound Repair

The process of reepithelialization is critical for completing wound healing. The epithelial cells from wound edges and dermal appendages proliferate and migrate across the wound and finally form a barrier between the wound and environment. Emerging evidence indicates that ROS from Nox and other resources regulate epithelial cells proliferation. In physiological condition, Nox1 is mainly present in large intestine epithelial cells, with much lower expression in the small intestine (jejunum, ileum) and the uterine and prostate [29]. However, constitutively expression of Nox1 and cytosolic proteins Rac1, p40^phox^, and p67^phox^ was identified as a source of superoxide in human immortalized skin (HaCaT) and gingival mucosal (GM16) keratinocyte cell lines [22]. Although Nox2 and Nox4 mRNA levels were also detected in both cell lines, Nox1 but not Nox4 protein was detected in HaCaT and GM16 cells, indicating that Nox1 may play a vital role in redox-mediated signaling which is associated with wound healing after tissue injury and epithelial tumorigenesis in human keratinocytes [22].

Rapidly increased NADPH oxidase activity and intracellular ROS were also found on human keratinocytes after treatment with a nontoxic dose of UVA radiation, which is a major environmental stress on skin [17]. Depleting the Nox1 isoform of the catalytic subunit of NADPH oxidase using small interfering RNA (siRNA) blocked the UVA-induced ROS increase and UVA-initiated prostaglandin E_2_ (PGE_2_) synthesis [17]. The increase in intracellular calcium was suggested to induce the activation of Nox1 [17]. Nox1 subunit of NADPH oxidase was also demonstrated to be of importance for UVA-induced ROS and PGE_2_ production, which might cause photosensitivity to UVA in patients with Smith-Lemli-Opitz syndrome (SLOS) [30]. These results indicate that UVA activates Nox1-based NADPH oxidase to produce ROS that stimulate PGE_2_ synthesis and that Nox1 may be an appropriate target for agents designed to block UVA-induced skin injury and tumor promotion. And another in vitro experiment indicated that Nox1 lies downstream of BLT2 and mediates UVB-induced ROS production and apoptosis of HaCaT [31]. This was further testified by in vivo BLT2-blocking and -overexpressing animal models, which concluded that “BLT2-Nox1”-linked pathway plays a crucial role in UVB-induced ROS generation and mediates apoptosis in human keratinocytes [31].

Using scratch wound motility assay, Nox1 and Nox4 isozymes of NADPH oxidase involved in enhanced ROS production and migration of HaCaT cells with cotreatment of hepatocyte growth factor (HGF) and transforming growth factor-*β*1 (TGF-*β*1) [19]. Reepithelialization by means of proliferation and migration of keratinocytes from the margin is one of the principal events in the process of wound healing. HGF and TGF-*β*1 as well as other various growth factors accelerate tissue repair by enhancing proliferation and migration of keratinocytes, fibroblasts, and endothelial cells and promoting the formation of granulation tissue [32, 33]. Another study testified that growth factor neuregulin, a member of the epidermal growth factor family (EGF), could also activate ROS generation through Nox1 and Nox2 and further increase cofilin dephosphorylation and activation in HaCaT keratinocytes to promote cell migration [34]. Thus, Nox1 might be beneficial in the process of tissue repair by participating in growth factor induced enhancement of reepithelialization. However, Nox1-generated ROS was found to mediate EGF-induced inhibition of the Rho activity in human colon cancer Caco2 cells that may be required for cell migration [35].

Like growth factor, other cytokines such as inflammatory factor signal transduction also have Nox1 and ROS involved in epithelial cells. Nox1 is responsible for the rapid production of ROS in response to IL-13 and Interferon-*γ* stimulation in human intestinal epithelial cells [36, 37]. Nox1 and ROS respond to IL-13 treatment, regulate phosphorylation of ERK1/2 and STAT6, and further increase the expression of intestinal trefoil factor 3 (TFF3) and antiapoptosis factor Bcl-xl, which contributes to the epithelial restitution and wound healing [36]. Interestingly, Nox1 may also induce the increased expression of the homologues Nox4 and Duox2 in IL-13 treated intestinal epithelial cells [36].

Nox1 was demonstrated to render immortalized human gingival mucosal keratinocytes resistant against Ca^2+^/serum induced differentiation [38]. Nox1-transfected cells produced fast dividing resistant cells and contained varying amounts of vimentin and K8/K18, which are associated with malignant progression in different types of human epithelial tumors [38]. And Nox1 was measured to be expressed in colon cancer samples and cancer cell lines Caco2, HT29, and T84 and human melanoma cell lines, which may likely be able to facilitate cancer cell migration and invasion [29, 39, 40]. This makes us concerned about the fact that Nox1 may be one of the crucial signal sources triggering the formation of epithelial tumor in people experiencing too much UV radiation exposure and patients suffering chronic wounds. However, evidence was also presented that Nox1 is not a mitogenic oxidase and suggests that Nox1 functions as a specialized phox-like enzyme in differentiated colon epithelium [29].

Beside intestinal epithelial cells and skin keratinocytes, Nox1 is likely to be the key source of ROS in lung epithelial cells after hypoxia injury and influenza virus infection [18, 41]. All of these confirm the crucial role of Nox1 in physiological homeostasis and pathological development of epithelial tissue, including defense bacterial invasion and radiation injury of intestinal and skin epithelium, promoting cell proliferation and migration in wound healing and tumorigenesis.

## 4. The Effects of Nox1 and ROS on ECM Deposition and Fibrosis

Fibroblast and myofibroblast cells, the main sources of extracellular matrix (ECM), especially collagen fibers, are indispensable in wound repair. Nox1 as well as Nox4 and Nox5 are constitutively expressed in human corneal stromal fibroblasts in mRNA level, contributing to the main source of superoxide [42]. The transcription of Nox1 mRNA in mouse embryonic fibroblasts (MEFs) could be elevated by calcium ionophore ionomycin in a dose-dependent manner [43]. Constitutively expressed or induced expressed Nox1 in fibroblast cells and Nox1-dependent superoxide may be potential for regulating gene expression and participating in the processes of inflammation and wound repair, as second signal messengers.

NIH3T3 cells overexpression of mox1 (also known as Nox1) was shown to increase superoxide generation and cell growth [15]. The transfected cells also had a transformed appearance, showed anchorage-independent growth, and produced tumors in athymic mice [15]. In another study, Nox1-generated oxidants were shown to downregulate the Rho activity through inactivation of the low molecular weight protein-tyrosine phosphatase in K-Ras-transformed normal rat kidney fibroblast cells, which leads to disruption of both actin stress fibers and focal adhesions [35]. In addition, fibroblast Nox1 was also demonstrated to form a complex with TRADD, RIP1 and Rac1, and be responsible for TNF-induced superoxide generation in murine fibrosarcoma L929 cells and MEF cells [44]. The large number of superoxides caused by adding TNF to these cells induced prolonged c-jun N-terminal kinase (JNK) activation and finally led cells to die, while knockdown of Nox1 using siRNA inhibited cell necrosis [44].

The fibrotic factors TGF-*β* and fibronectin were induced to express much more in diabetes milieu through the interaction of Nox1 and iNOS (inducible nitric oxide synthase) [45]. The increased expression of fibrotic factors is suggested to be one of key mechanisms for kidney fibrogenesis of diabetes patients. The NADPH oxidase inhibitor and antioxidant reduced the expression of profibrotic factor TGF-*β* and collagen accumulation in cultured glomerular mesangial cells [46, 47]. And in vivo studies suggested that interventions with various antioxidants or NADPH oxidase inhibitor apocynin have beneficial effects on renal fibrosis [48, 49]. However, there is no direct or indirect evidence indicating that Nox1 rather than other Nox family members plays crucial role in renal fibrosis.

The matricellular protein CCN1 (also known as CYR61: cysteine-rich protein 61), which regulates diverse cellular functions, including cell adhesion, migration, differentiation, and survival in a cell-type and context-dependent manner, is dynamically expressed at sites of wound repair [50]. CCN1, highly expressed in granulation tissues during cutaneous wound healing, drives fibroblasts into senescence and upregulates the expression of antifibrotic genes to restrict fibrosis during tissue wound repair [51, 52]. It could induce fibroblast senescence by binding to integrin *α*6*β*1 and the heparan sulphate proteoglycans, which further induces DNA damage response pathways and activates p53 and the Rac1-Nox1 complex. CCN1 is unique among ECM proteins as a cell-adhesive substrate in triggering a robust and sustained accumulation of ROS necessary for senescence. The activated Nox1 results in the ROS-dependent activation of the p16^INK4a^/pRb pathway, leading to senescence and concomitant expression of antifibrotic genes [52]. Therefore, CCN1-Nox1 dependent fibroblast senescence response in cutaneous injury functions to curb fibrosis during wound healing.

## 5. Nox1 Regulates Vessel Damage and New Vessel Formation

Endothelial cells and smooth muscle cells of vascular system play a central role in angiogenesis and vascular remodeling during tissue injury, and repair, including ischemic heart disease, peripheral artery disease, acute lung injury, and wound healing. Angiogenesis, the process of new blood vessel growth, is dependent on cell proliferation, migration, and capillary tube formation, which is partially regulated by the redox system. The effects of ROS on vascular cells are tightly regulated and dependent on the amount and site of production as well as the intracellular balance of prooxidant and antioxidant enzyme activity. Low levels of ROS appear to be physiological and beneficial cellular signal in reparative angiogenesis in response to ischemia and wound healing [6, 53], while excess amount of ROS contributes to endothelial cells and smooth muscle cells injury and dysfunction [54]. In the vascular system, Nox1 mRNA has been detected mainly in vascular smooth muscle cells but not in adventitial cells, whereas Nox2 is localized primarily in endothelial and adventitial cells, and Nox4 is abundantly expressed in all of the vessel constituents [55–57]. And more recently, all Nox isoforms (Nox1–5) were shown to exist in human cardiovascular cells, and the whole-cell and nuclear levels of Nox1 were reported to be similar in human vascular endothelial cells and smooth muscle cells [58]. Nox1 was suggested to be strongly related to vascular physiopathological changes, such as angiotensin II-induced hypertension and aortic dissection [59, 60] and atherosclerosis development in diabetic apolipoprotein E-deficient mice [61].

Vascular endothelial growth factor (VEGF) and its receptor VEGFR play an indispensable role in angiogenesis after tissue injury. VEGF, a potent angiogenic growth factor, primarily through VEGFR2 (KDR/Flk1), stimulates proliferation, migration, cytoskeletal reorganization, and tube formation of endothelial cells. VEGFR is activated through dimerization and autophosphorylation of tyrosine residues in the cytoplasmic kinase domain [62]. The receptor activation is followed by activation of downstream signaling pathways such as mitogen-activated protein kinases, PI3 kinase, Src, Akt, and eNOS, which are essential to induce endothelial cell migration and proliferation and contribute to angiogenesis [54, 62, 63]. NADPH oxidase is one of the major sources of ROS in endothelial cells. VEGF can activate NADPH oxidase and induce the production of ROS, which in turn can increase the expression of VEGF and VEGFR and be involved in VEGFR phosphorylation, cell proliferation, and migration [64, 65]. The major producers of ROS in endothelial cells have been thought to be Nox2 and Nox4, as well as xanthine oxidase and eNOS, rather than Nox1 [66]. However, Nox1 was found to be expressed in activated sinusoidal endothelial cells (NP31). The expression of Nox1 mRNA was increased by approximately sixfold in NP31/kinase cells (transformed by the introduction of a constitutively activated form of the VEGFR1 kinase) by Northern blotting [67]. Neither Nox2 expression in NP31 cells nor induced expression of Nox2 in transformed Np31/kinase cells was detected [67]. Anti-Nox1 siRNA treatment failed to inhibit tubulogenesis in vitro, indicating that Nox1 together with Ras and other molecules participated in the VEGFR1 kinase-derived tubulogenic pathway through ROS [67].

The overexpression of Nox1 and hydrogen peroxide from NIH 3T3 cells as well as DU-145 cells was demonstrated to trigger the conversion of previous dormant tumors to the angiogenic phenotype, which indicates the progress of dormant tumors to active tumors. Nox1 led to a nearly 10-fold increase in hydrogen peroxide levels, 4-fold induction of VEGF mRNA in NIH 3T3 cells, and high-level expression of VEGFR1 and VEGFR2 in the newly growing blood vessels [65]. Zymographic analysis also showed that Nox1 expression induced increment of matrix metalloproteinase MMP-9 bioactivity [65]. The balance of protease and antiprotease activity in the vascular tissue is thought to play an important role in pathogenesis of aortic dissection and aneurysm as well as the process of angiogenesis. Matrix metalloproteinases are thought to favor the pathogenesis of vascular disease and promote new blood vessels formation, whereas the specific inhibitor tissue inhibitor of metalloproteinases (TIMPs) is thought to be preventive. In a hypertension animal model, angiotensin II increased TIMP-1 mRNA level in both Nox1 deficient mice and wild type mice but increased TIMP-1 mRNA protein levels much more tremendously in Nox1 deficient mice than wild type mice [60]. This indicated that Nox1 might be able to alter the protease and antiprotease equilibrium, which is critical for vascular tissue injury and wound healing.

Besides endothelial cells, vascular smooth muscle cells participate in new vessel development through cell migration, proliferation, and extracellular matrix production after tissue injury. Nox1 and its oxidant were shown in vitro to contribute to vascular smooth muscle cell proliferation and neointima formation induced by urokinase plasminogen activator (uPA) [68]. Basic fibroblast growth factor (bFGF) induced migration of vascular smooth muscle cells is mediated by Nox1 rather than Nox4, through phosphorylation of the adaptor protein paxillin, which is essential for migration and secretion of MMPs [16]. And an in vivo animal study demonstrated that Nox1 and Nox2 from medial and neointimal smooth muscle cells and adventitial fibroblasts contributed to the increased superoxide production 3 to 15 days after balloon injury of the rat carotid artery [69]. The activated Nox1 and oxidative stress may be critical to smooth muscle phenotypic modulation in restenosis. Furthermore, wire injury-induced neointima formation in the femoral artery, along with proliferation and apoptosis, was reduced in Nox1 knock out mice [70]. Compared to wild type cells, in vitro cultured Nox1 knock out smooth muscle cells exhibited more phosphorylated cofilin (a regulator of actin depolymerization) both basally and after PDGF stimulation, without alteration of cofilin expression [70]. Phosphorylation of cofilin at Ser3 inhibits its activity, which is responsible for reduced migration of Nox1 deficient cells [70].

Nevertheless, it seems that Nox1 also can inhibit endothelial cell proliferation. Chronic treatment primary human umbilical vein endothelial cells with resveratrol (10 *μ*M) elevated ROS levels (mainly from Nox1 and Nox4) that were linked to an accumulation of cells in S phase [71]. This indicates that Nox1 and other Nox proteins in vascular system have a complex regulation mechanism under different conditions, which needs specific research for targeting therapy.

## 6. Nox1 and ROS from Other Cells Involving Cell Damage and Dysfunction

As mentioned above Nox1 is broadly expressed in various cell types besides epithelial cell, endothelial cell, and vascular smooth muscle cell. Nox1 is expressed in BeWo choriocarcinoma cells, which can be activated by EGF [72]. This is further confirmed by the evidence that in placental tissues Nox1 was localized in syncytiotrophoblasts, in villous vascular endothelium, and in some stromal cells, which is increased in patients with preeclampsia [72]. Additionally, expression of Nox1 protein and ROS is increased in pancreatic beta cells in response to proinflammatory cytokines stimulation, which might lead to cell dysfunction and death [73]. Moreover, Nox1 is suggested to play critical roles in spermatogonial stem cells self-renewal via the activation of the p38 MAPK and JNK [74].

## 7. Nox1 and Inflammation

Inflammation following injury is the essential process for tissue repair. Inflammatory cells such as neutrophils and macrophages are major source of ROS, which are needed for scavenging bacteria and necrotic tissue. As mentioned above, the activation of Nox1 and rapid production of ROS could be induced by inflammatory cytokines like IL-13 and interferon-*γ* [36, 37]. Nox1 and its derived ROS further participate in intracellular signaling processes regulating gene expression, which contributes to cell proliferation, differentiation, and tissue repair.

ROS may serve as the primary signal inducing the migration of inflammatory cells directly after tissue injury. In a study performed on zebrafish larvae, a rapid and sustained increase of hydrogen peroxide at the wound margin was detected upon local injury of the tail fin, which occurred before the recruitment of leukocytes, suggesting that the source of hydrogen peroxide was the tail fin epithelium, not leukocytes [13]. This finding contrasts with the prevailing view that the ROS molecules found at the wound site are mainly produced by oxidative bursts of inflammatory cells. Furthermore, Duox1 in epithelium was shown to be the Nox isoform responsible for the early ROS production after epithelial injury [13]. As of now, there is no evidence indicating that local Nox1 can be activated upon tissue injury and form a ROS gradient activating migration of inflammatory cells, as its homologue Duox1 did in zebrafish. However, Nox1 and its derived ROS can indirectly affect inflammation by regulating inflammatory cytokines such as PGE_2_, CCL2, CCL3, CXCL2, IL-1*β*, GM-CSF, and TNF-*α* [17, 30, 41].

## 8. Conclusion and Future Perspectives

NADPH oxidase, whose only function is ROS production, has been extensively investigated in many cell types of both mammalian and plant. Nox1, one member of the NADPH oxidase family, is not only constitutively expressed in a variety of tissues, but also induced to increase expression in both mRNA and protein levels under many circumstances. Nox1 is most expressed by intestinal epithelium and participates in the maintenance of epithelial barrier and mucosal homeostasis, including promoting intestinal mucosa wound healing by activation of focal cell matrix adhesion proteins and cell motility. It could also be induced and activated by growth factors, inflammatory cytokines, and UV injury in skin and mucosal keratinocytes in vitro, regulating cell proliferation, differentiation, and migration. In addition, Nox1 and ROS have been suggested to regulate cellular differentiation, ECM deposition, and fibrosis formation both in vitro and in vivo. As for in the processes of blood vessel damage and regeneration, Nox1 from smooth muscle cell and endothelial cell, together with some of its homologues (Nox2 and Nox4), is critical for vascular injury response and pathology. Nox1 and its derived ROS are also involved in inflammatory response caused by injury or infection through interaction with inflammatory cells. In short, Nox1 and its derived reactive oxygen species are crucial intracellular signaling regulators involving cell proliferation, differentiation, migration, extracellular matrix production and deposition, and inflammatory process after tissue injury, which contribute to the processes of tissue repair (Table 1).

Although a large number of evidence indicates that Nox1 has critical effect upon the process of tissue repair, there are many open questions regarding the complex mechanisms of Nox1. Nox1, as well as some of its homologues, has broad tissue and cellular distribution and is either constitutively expressed or stimulatively upregulated. One tissue or cell may express various NADPH oxidase molecules, and the same cell type in different tissues may have different dominant Nox isoforms at mRNA and protein levels [7, 15, 55–57]. Nox molecules are activated by different stimuli and present the function of proinjuring or protection [41, 75]. Cross talk exists between Nox homologues so that the activation of one Nox might further activate other Nox molecules in the same cell through the production and regulation of ROS [36, 76]. Additionally, Nox1 accomplishes its functions through complicated and unique intracellular signaling pathways upon disparate stimuli. Therefore, in order to get improved understanding of the roles of Nox1 in the process of tissue repair, the specificity of cell and tissue, the type of injury and severity of injury, the specific intracellular signaling, and the potential cross talk between various Nox homologues remain to be elucidated.

## Figures and Tables

**Table 1:** Nox1 expression, intracellular signaling, and function.

Cells involved	Intracellular signaling	Function	References
Intestinal epithelial cell; colon carcinoma cells (Caco2 and HT29)	IL13-Nox1-ERK/STAT6-TFF3/Bcl-xl; Nox1-RhoA-alpha3 integrin	Proliferation and differentiation; migration	[29, 36, 37, 40]
HaCaT; GM16	Ca^2+^-Nox1-PGE_2_; Ca^2+^/serum-Nox1-vimentin/K8/K18	Proliferation and host defense; skin injury; apoptosis; migration	[17, 19, 22, 30, 31, 34, 38]
Lung epithelial cell	Nox1-JNK/ERK/Caspase-3	Cell death and protection	[18, 41]
Corneal stromal fibroblast		May participate in inflammation	[42]
Mouse embryonic fibroblast		Ischemia-reperfusion injury	[43]
NIH 3T3 fibroblast; rat kidney fibroblast	Ras-Nox1-Rho-actin stress fibers and focal adhesions	Tumorigenic conversion	[15, 35]
Fibrosarcoma L929 cells	TNF-TRADD/RIP1/Rac1/Nox1	Necrosis	[44]
Human BJ foreskin fibroblast and IMR-90 lung fibroblast	CCN1-Nox1/Rac-ERK/p38 MAPK-p16^INK4a^/pRb	Senescence and expression of antifibrotic genes	[52]
Mouse vessel	Angiotensin II-Nox1-nitric oxide	Hypertension	[59]
Mouse aorta	Angiotensin II-Nox1-tissue inhibitor of metalloproteinase 1	Aortic dissection	[60]
Human aortic endothelial cell	Hyperglycemia-Nox1-proinflammatory and profibrotic markers	Atherosclerosis	[61]
Rat kidney cell; tumor cells and vessel; sinusoidal endothelial cell	K-Ras-Nox1-ERK-sp1-VEGF; Nox1-VEGF/VEGFR/MMP	Upregulate VEFG expression; increase tumorigenicity and upregulate VEGF/VEGFR and MMP; tubulogenic	[64, 65, 67]
Vascular smooth muscle cell	uPA-Nox1/Nox4; PDGF-Nox1-cofilin	Migration; proliferation and necrosis	[68–70]
